# Functional Validation of a Novel Homozygous *TTN* Splice‐Site Variant Reveals Aberrant Splicing in Hypertrophic Cardiomyopathy

**DOI:** 10.1155/humu/4229370

**Published:** 2026-09-03

**Authors:** Xiaoyun Sun, Dandan Wang, Shujuan Wang, Rong Wan, Zhenhong Jiang, Wenqiang Zhang, Jin Ouyang, Yang Shen, Kui Hong

**Affiliations:** ^1^ Department of Medical Genetics, The Second Affiliated Hospital of Nanchang University, Nanchang, China, jxndefy.cn; ^2^ Department of Cardiovascular Medicine, The Second Affiliated Hospital of Nanchang University, Nanchang, China, jxndefy.cn; ^3^ Jiangxi Province Key Laboratory of Molecular Medicine, The Second Affiliated Hospital of Nanchang University, Nanchang, China, jxndefy.cn

**Keywords:** genetic testing, hypertrophic cardiomyopathy, *TTN*, variant interpretation

## Abstract

The *TTN* gene encodes a crucial structural protein within cardiac sarcomeres, and its variants may contribute to hypertrophic cardiomyopathy (HCM) and dilated cardiomyopathy; however, phenotype and genotype are different. Whole‐exome sequencing (WES) was conducted on a Chinese proband diagnosed with HCM. In silico splicing prediction tools and minigene assays were employed to investigate the impact of the identified variant on mRNA splicing. A literature review was performed to retrieve and analyze previously reported splicing variants in the *TTN* gene associated with HCM. A 42‐year‐old male proband presented with nonobstructive HCM and paroxysmal atrial arrhythmias. A novel homozygous *TTN* variant was found, predicted to cause a 14‐base pair deletion at a splice acceptor site. Two asymptomatic offspring were found to carry the heterozygous variant. Based on variant interpretation guidelines, the variant met the PM2_supporting criterion and was classified as a variant of uncertain significance (VUS). The predicted aberrant splicing effect was subsequently confirmed by the minigene splicing assay, demonstrating altered pre‐mRNA splicing leading to an in‐frame insertion/deletion (p.Arg32498_Glu32504delinsGln). Functional verification confirmed that this mutation conforms to the PM4 criterion, suggesting that it can be reclassified as a tepid VUS (scoring 3 points). The functional result might provide pathogenic evidence. We also reviewed 28 previously reported splicing variants in *TTN* associated with HCM. Of these, 57.1% (16/28) localized to the I‐band region, whereas 21.4% (6/28) were situated in the A‐band domain of titin. Notably, 21.4% (6/28) co‐occurred with pathogenic variants in other sarcomeric genes (*MYH7* or *MYBPC3*), correlating with more severe clinical phenotypes. Reclassification and reinterpretation of the variants revealed that none met the level of likely pathogenic or higher. The present case contributes a homozygous splice‐site variant with experimentally confirmed aberrant splicing and an in‐frame protein alteration in titin. The focus of this report is the Mendelian genetic basis of the proband′s cardiomyopathy phenotype, and our data provide additional case‐level and functional evidence for a possible role of specific *TTN* splicing defects in HCM.

## 1. Introduction

Hypertrophic cardiomyopathy (HCM) is the most prevalent inherited cardiomyopathy, characterized by left ventricular hypertrophy that is not solely explained by hemodynamic load. Current estimates indicate a prevalence of approximately 1 in 200 individuals [1]. HCM is typically inherited in an autosomal dominant (AD) pattern with incomplete penetrance. Approximately 60% of patients with HCM harbor pathogenic variants, primarily in genes encoding myocardial sarcomere proteins [2, 3]. Variants in *MYH7* and *MYBPC3* represent the most frequent genetic etiologies, accounting for over 50% of cases, followed by *TNNT2* and *TNNI3* [4–7]. The Clinical Genome Resource (ClinGen) categorizes HCM‐associated genes based on the strength of supporting evidence. Genes such as *MYH7*, *MYBPC3*, *TNNT2*, *TNNI3*, *TMP1*, *MYL2*, *MYL3*, and *ACTC1* are classified as having “definitive” evidence, whereas *TTN* is currently categorized as having only “limited” evidence [8].

Titin is a large, multidomain protein crucial for sarcomere structural integrity and passive stiffness, modulating active contractile force generation [9, 10]. It consists of repeating immunoglobulin (Ig) and Fibronectin type III (FN‐III) modules interspersed with nonrepetitive sequences that contain phosphorylation sites, PEVK motifs, and a terminal kinase. Alternative splicing of *TTN* pre‐mRNA generates several cardiac isoforms, including N2B, N2BA, and Novex‐3 [11]. *TTN* is the most common definitive pathogenic gene in dilated cardiomyopathy (DCM), accounting for 25% of cases [12, 13]. Importantly, *TTN* has also been identified as a candidate gene for HCM, left ventricular noncompaction cardiomyopathy (LVNC), and arrhythmogenic right ventricular cardiomyopathy (ARVC) [14–16]. *TTN*‐truncating variants (TTNtv), especially those enriched in the A‐band region, are known pathogenic drivers of DCM. These typically include frameshift, nonsense, and essential splice‐site variants [13, 17]. In contrast, the association between *TTN* and HCM is supported by limited and inconclusive evidence.

In this study, we report a patient with nonobstructive HCM and atrial fibrillation (AF)/flutter carrying a novel homozygous *TTN* splicing variant and investigate its effect on pre‐mRNA splicing using a minigene assay. Our findings aim to enhance a more comprehensive understanding of the role of this specific *TTN* variant, thereby informing the ongoing evaluation of *TTN*′s contribution to the genetic basis of HCM.

## 2. Materials and Methods

### 2.1. Clinical Evaluation and Ethical Compliance

A 42‐year‐old male patient presented with recurring chest tightness and palpitations, subsequently receiving medical care at The Second Affiliated Hospital of Nanchang University. Various clinical diagnostic examinations were conducted, including electrocardiogram (ECG), transthoracic echocardiography (TTE), computed tomography (CT) and laboratory tests. The patient was prescribed metoprolol. The Ethics Committee of the Second Affiliated Hospital of Nanchang University approved this study, and we obtained the patient′s written informed consent.

### 2.2. Whole Exome Sequencing (WES)

Genomic DNA was extracted from a peripheral blood sample of the patient. All steps of WES were performed by the Department of Medical Genetics of The Second Affiliated Hospital of Nanchang University in Jiangxi, China. The process included genomic DNA extraction, exome library preparation, exome capture, and sequencing on the Nextseq 550DX platform (Illumina, United States) following alignment to the human reference genome (GRCh37/hg19), variants such as single nucleotide variants (SNVs), insertions and/or deletions (indels), and others were detected using Dynamic Read Analysis for GENomics software, with annotation performed using relevant databases. Finally, variant validation and cosegregation analysis were confirmed by Sanger sequencing in the core family. The sequences of the primer were 5 ^′^‐GCACATGTACCCCTGAAGCT‐3 ^′^ and 5 ^′^‐ATCCATGGCAACGATGGGTT‐3 ^′^. The 3500DX XL platform (Life, United States) was employed for this purpose.

### 2.3. Bioinformatics Analysis

WES was performed for the proband. Variant analysis was initially focused on a virtual gene set of cardiomyopathies‐ and inherited arrhythmia‐associated genes with ClinGen‐curated or ClinGen‐based gene‐disease validity classifications of limited or higher. The genes included in this analysis, together with their associated phenotypes and evidence classifications, are listed in Table S2. Multiple online in silico splice‐site predictions were used to evaluate the potential pathogenicity of the splicing variants, including RDDC^SC^ (https://rddc.tsinghua-gd.org/), VarSEAK (https://varseak.bio/index.php), and SpliceAI (https://spliceailookup.broadinstitute.org/). These predictions suggested potential aberrant splicing caused by the identified variant (seen in the Figure S1).

### 2.4. The Minigene Splicing Assay

**Figure 1:**
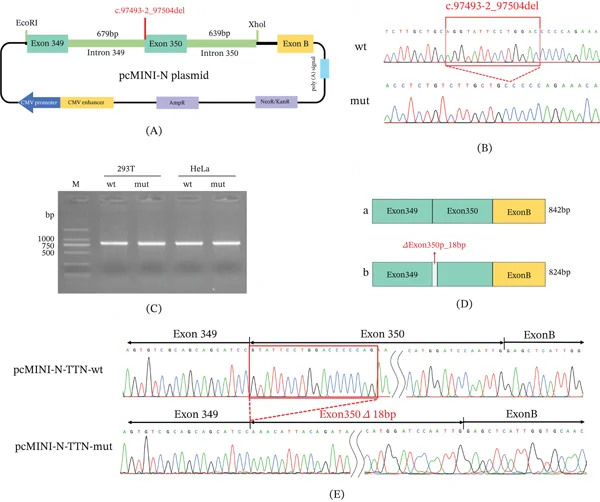
The effect of *TTN* c.97493‐2_97504del variant on splicing. (A) Schematic diagram of vector construction used in minigene assay. (B) The Sanger sequencing of the minigene plasmid shows the successful site‐directed mutagenesis of the variant, wt, wild type; mut, mutant. (C) Agarose gel electrophoresis for RT‐PCR analysis, bands in HeLa and 293T cells are labeled a and b, respectively, M indicates the ladder. It shows a qualitative RT‐PCR analysis of minigene‐derived transcripts. Band intensity was not used for quantitative comparison of transcript abundance. (D) Schematic diagram of the minigene plasmid showed 18‐bp deletion in Exon 350. (E) Sanger sequencing of the transcript of the minigene plasmid showed 18‐bp deletion in Exon 350.

The minigene assay had been extensively validated in previous studies and was considered an effective, reliable, and relatively simple tool for evaluating potential RNA splicing alterations. We used the pcMINI vector that we developed previously, which contained a multiple cloning site (Figure 1A). We identified the genomic region surrounding the c.97493‐2_97504del variant and designed primers to amplify this region from the genomic DNA of controls using DNA Polymerase‐PrimeSTAR MAX DNA Polymerase (R045A, TaKaRa, Japan). Subsequently, the PCR products were cloned into pcMINI vectors using EcoRI/XhoI digestion and a recombination reaction. Mutant constructs containing the c.97493‐2_97504del variant were generated by PCR using the mutant primers (Method S1 and Table S1). The constructed vectors were then transformed into Escherichia coli DH5*α*‐competent cells and selected for PCR amplification. Following direct confirmation of wild type/mutant (wt/mut) plasmids by Sanger sequencing, both types of plasmids were transfected into HEK293T and HeLa cells. After 48 h, total RNA was extracted using the RNA extraction kit (Trizol RNAiso PLUS, 9109, TaKaRa, Japan) and reverse transcribed with Hifair 1st Strand cDNA Synthesis SuperMix for qPCR (gDNA digester plus) (11123ES70, YEASEN, China). The resulting cDNA was then PCR‐amplified. Finally, the amplified products were analyzed through 1.5% agarose gel electrophoresis, purified with the DNA Gel Extraction Kit (2001250, SIMGEN, China), and subsequently sequenced by Sanger sequencing.

## 3. Result

### 3.1. Clinical and Follow‐Up Results

**Figure 2:**
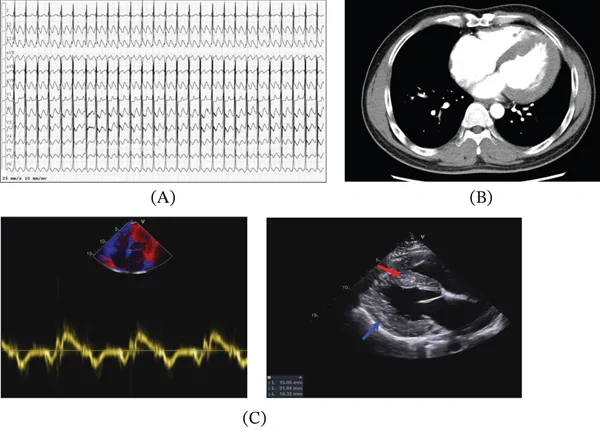
The clinical analysis of the proband. (A) The 12‐lead electrocardiogram (ECG) shows 2:1 atrial flutter. (B) Left atrium CT suggests mild enlargement of the left atrium, myocardial hypertrophy of the left ventricle, and pericardial effusion. (C) The echocardiographic image shows symmetrical nonobstructive left ventricular (LV) hypertrophy. The red arrow indicates the interventricular septum with 15–22 mm, and the blue arrow indicates the left ventricular free wall with 19 mm.

The 42‐year‐old male patient presented with recurrent chest tightness and palpitations. The patient exhibited clear consciousness and clear breath sounds in both lungs. Heart rate was elevated at 168 beats per minute with arrhythmia noted, characterized by unequal intensity of the first heart sound, and the intensity of the second heart sound in the aortic valve area was greater than in the pulmonary valve area. No other abnormal physical signs were observed. The proband did not report skeletal muscle weakness, myalgia, recurrent falls, respiratory muscle involvement, or other neuromuscular symptoms, and no obvious skeletal muscle abnormalities were documented during routine physical examination. The proband had no severe valvular disease, thyroid dysfunction, ischemic heart disease, diabetes mellitus, hypertension, or obesity. However, he had a history of smoking, which may represent a potential risk factor for AF/flutter [18]. ECG revealed paroxysmal atrial flutter (Figure 2A). TTE confirmed a diagnosis of nonobstructive HCM with preserved left ventricular ejection fraction (58%) (Figure 2C). Chest CT showed mild left atrial enlargement, left ventricular hypertrophy, and pericardial effusion (Figure 2B). Twenty‐four–hour ECG monitoring detected episodes of paroxysmal AF and supraventricular tachycardia. Limb peripheral nerve electromyography showed no characteristic abnormalities of peripheral nerves or skeletal muscles, and muscle MRI revealed no abnormal findings. HCM and AF/flutter were recognized at the same clinical presentation during this hospitalization. After further evaluation, the patient was diagnosed with HCM and paroxysmal AF. Clinical evaluation of the patient′s family members, including his parents, wife, 18‐year‐old son, and 14‐year‐old daughter, revealed they were asymptomatic. Based on the available family history, no known consanguinity was reported in the proband′s parents or wider family.

To manage the condition, the patient was treated with metoprolol for ventricular remodeling and rivaroxaban as anticoagulant therapy. The follow‐up results showed a persistently hypertrophic interventricular septum (IVS) and a reduction in the thickness of the left ventricular posterior wall (LVPW) and the diameter of the left atrial diameter (LAD) (Table 1).

**Table 1:** Follow‐up TTE results of the proband.

Time	IVS (mm) (8–11)	LVPW (mm) (8–11)	LVEF (%) (50–70)	LAD (mm) (< 35)
2022/03	15–22	19	58	53
2022/09	14–23	17	55	46
2023/07	12–24	18	56	43
2024/12	12–23	14	57	43

Abbreviations: IVS, interventricular septum thickness; LAD, left atrial diameter; LVEF, left ventricular ejection fraction; LVPW, left ventricular posterior wall.

### 3.2. Identification of a Homozygous TTN Splice‐Site Variant

**Figure 3:**
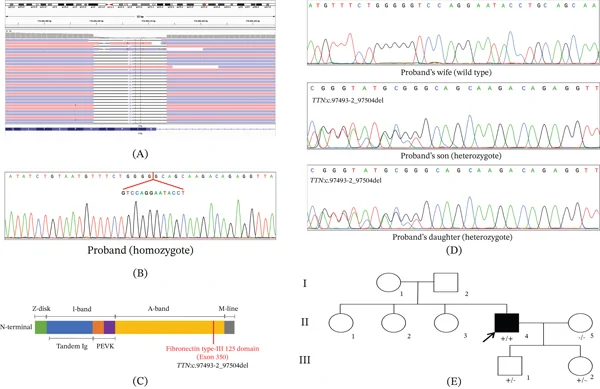
The genetic analysis of proband and cascade family screening. (A) Integrated genome view of the *TTN* variant (chr2: g.179406300_179406313del) with IGV software. (B) The Sanger sequencing data of the variant in the proband. (C) Schematic representation of the fibronectin type‐III 125 domain affected by the splice variant in titin. (D) The Sanger sequencing data of the variant in the proband′s wife and children. (E) The pedigree of the family. The arrow indicates the proband; the black square indicates the male patient; +/+ indicates the homozygous variant; +/− indicates the heterozygous variant carrier; and −/− indicates the wild type.

WES identified a novel homozygous 14‐base pair deletion in the *TTN* gene (chr2: g.179406300_179406313del; NM_001267550.2: c.97493‐2_97504del) in the proband (Figure 3A), which was subsequently confirmed by Sanger sequencing (Figure 3B). The variant involves the canonical splice acceptor region of *TTN* meta‐transcript Exon 350, corresponding to LRG Exon 351 according to CardioDB/LRG exon annotation. This exon is located within the A‐band region of titin and encodes the FN‐III 125 domain (Figure 3C). The affected exon is highly included in cardiac *TTN* transcripts, with percentage spliced‐in (PSI) values of 100% in DCM left ventricular tissue and 98% in GTEx left ventricular tissue. No additional rare pathogenic or likely pathogenic (LP) variants in established HCM‐associated genes or major cardiomyopathy/arrhythmia‐related genes were identified after variant filtering; the remaining variants were classified as benign (B) or likely benign (LB) or were considered clinically unrelated. Cascade genetic screening via Sanger sequencing showed the proband′s wife was wild‐type at this locus, whereas his asymptomatic 18‐year‐old son and 14‐year‐old daughter were heterozygous for the variant (Figure 3D). The family pedigree is illustrated in Figure 3E.

### 3.3. Effect of the TTN c.97493‐2_97504del Variant on Splicing

Prediction tools suggested that the c.97493‐2_97504del variant disrupts the splice acceptor site. Specifically, SpliceAI (*Δ*score 0.99) predicted activation of a cryptic acceptor site. However, bioinformatic predictions alone were insufficient to establish the variant′s impact on RNA processing, in line with American College of Medical Genetics and Genomics and the Association for Molecular Pathology (ACMG/AMP) guidelines. To assess its functional consequence, we constructed a minigene vector containing _*TTN*_ Exon 349, Intron 349, and Exon 350, harboring the c.97493‐2_97504del variant (Figure 1A,B). Transfection of this construct into HEK‐293T and HeLa cell lines, followed by RT‐PCR analysis, revealed no exon skipping (Figure 1C). Sequencing of the cDNA revealed that the variant led to the utilization of a cryptic splice‐site located 18 bp downstream of the authentic acceptor site (Figure 1D,E). This aberrant splicing event resulted in the deletion of amino acids Arg32498‐Glu32504 and the insertion of a glutamine residue (c.97493_97510del; p.Arg32498_Glu32504delinsGln). Based on the ACMG/AMP variant interpretation criteria [19], the variant was classified as a variant of uncertain significance (VUS), supported by PM2_supporting and PM4 evidence codes.

## 4. Literature Review of *TTN* Splicing Variants in HCM

A comprehensive review of PubMed, ClinVar, and the Human Gene Mutation Database (HGMD) identified 28 reported *TTN* splicing variants associated with HCM (Table 2). These variants were re‐evaluated according to ACMG/AMP guidelines [19]. Of the analyzed variants, 57.1% (16/28) localized in the I‐band region, whereas 21.4% (6/28) were situated in the A‐band domain of titin. Notably, 21.4% (6/28) of the variants co‐occurred with pathogenic variants in sarcomeric genes (*MYH7* or *MYBPC3*), correlating with severe clinical phenotypes, such as unexplained syncope, sudden cardiac death, and nonsustained ventricular tachycardia. Of the 28 variants, 46.4% (13/28) were classified as VUS, underscoring the need for functional studies to clarify their role as disease modifiers or contributors to compound heterozygosity. In addition, 53.6% (15/28) variants were classified as LB or B. Most existing evidence is derived from familial case reports using next‐generation sequencing, which systematically excluded variants with high population frequency, localization to nonconstitutive exons, or co‐occurrence with other known pathogenic variants. However, few of these variants have been functionally validated using minigene assays or RNA sequencing.

**Table 2:** Summary of *TTN* splicing variants associated with HCM.

ID	Transcript	cDNA	Type	Intron	Cardiac PSI%	gnomAD (FAF)	Affected domain	SpliceAI prediction	Variants classification	Phenotypes	Co‐occurred variants	Reference
1	NM_001267550.2	c.1398+4C>T	Cryptic splicing	8	100	0.00023450	Z‐disk	△score < 0.20	LB	HCM	—	[20]
2	NM_001267550.2	c.22816+1G>T	Canonical splicing	78	35	NA	Ig‐like 54 (I‐band)	Donor loss:△score 0.98	VUS	HCM	—	[21]
3	NM_001267550.2	c.31763‐1G>A	Canonical splicing	122	78	0.00055920	—	Acceptor loss:△score 1; acceptor gain:△score 0.88	VUS	HCM	—	[13]
4	NM_001267550.2	c.34612+1G>T	Canonical splicing	150	20	NA	—	Donor loss:△score 0.99	VUS	HCM	*MYBPC3*:p.R495G	[22]
5	NM_001267550.2	c.35308+1G>T	Canonical splicing	156	5	NA	PEVK 5 (I‐band)	Donor loss:△score 0.98	VUS	HCM	—	[23]
6	NM_001267550.2	c.55432+5G>C	Cryptic splicing	286	100	0.00004868	Ig‐like 101 (A‐band)	△score < 0.20	VUS	HCM	*MYBPC3*: p. E474X	[15]
7	NM_001267550.2	c.59344+3G>A	Cryptic splicing	300	100	0.00174000	FN‐III 31 (I‐band)	△score < 0.20	LB	HCM, syncope, SCD	*MYBPC3*: c.1928‐2A>G	[15]
8	NM_001267550.2	c.98098+3G>A	Cryptic splicing	351	100	0.00111300	FN‐III 126 (A‐band)	△score < 0.20	LB	HCM, syncope, NSVT	*MYBPC3*:p.I807N	[15]
											*TNNT2*: p. I100M	
9	NM_001267550.2	c.106374+5C>A	Cryptic splicing	358	100	NA	Ig‐like 149 (M‐line)	△score < 0.20	VUS	HCM, LVOTO	*MYH7*: p. F46I	[15]
10	NM_001256850.1	c.28091‐2A>C	Canonical splicing	99	69	0.00127500	—	Acceptor loss:△score 1	LB	HCM	—	[20]
11	NM_001256850.1	c.34186+1G>T	Canonical splicing	152	48	NA	PEVK 15 (I‐band)	Donor loss:△score 0.98	VUS	HCM	—	[13]
12	NM_133378.4	c.10114+5G>A	Cryptic splicing	43	100	0.00923200	Ig‐like 20 (I‐band)	△score < 0.20	B	HCM	—	[23]
13	NM_133378.4	c.25310‐2A>C	Canonical splicing	97	72	0.00127500	Ig‐like 77 (I‐band)	Acceptor loss:△score 1	VUS	HCM	*MYH7*: p.R723C. *PKP4*: p.T319M	[24]
14	NM_133378.4	c.28031‐1G>A	Canonical splicing	118	78	0.00055920	PEVK 3 (I‐band)	Acceptor loss:△score 1	VUS	HCM	—	[24]
15	NM_133378.4	c.30094+4C>T	Cryptic splicing	140	74	0.00001154	PEVK 12 (I‐band)	△score < 0.20	VUS	HCM	—	[25]
16	NM_133378.4	c.32077+20C>G	Cryptic splicing	159	5	0.00000437	I‐band	△score < 0.20	LB	HCM	—	[26]
17	NM_133378.4	c.32407+6C>T	Cryptic splicing	163	7	0.00795300	PEVK 23 (I‐band)	△score < 0.20	LB	HCM, LQTS, SUD	—	[23]
18	NM_133378.4	c.43733‐2A>T	Canonical splicing	221	100	NA	I‐band	Acceptor loss:△score 1	VUS	HCM	—	[26]
19	NM_133378.4	c.49408‐4C>T	Cryptic splicing	241	100	0.00249700	FN‐III 26 (I‐band)	△score < 0.20	LB	HCM	—	[23, 25]
20	NM_133378.4	c.50143+6 T>A	Cryptic splicing	244	100	0.00558200	FN‐III 27 (I‐band)	△score < 0.20	LB	HCM	—	[23]
21	NM_133378.4	c.59066‐4A>T	Cryptic splicing	266	100	NA	Nonconstitutive	Acceptor loss:△score 0.09	LB	HCM	—	[26]
22	NM_133378.4	c.62012‐5C>G	Cryptic splicing	274	100	0.00373400	FN‐III 57 (A‐band)	△score < 0.20	LB	SUD, SUD/SIDS, HCM	—	[23]
23	NM_133378.4	c.80003‐4G>T	Cryptic splicing	277	100	0.00274100	FN‐III 101 (A‐band)	△score < 0.20	LB	HCM	—	[23]
24	NM_133378.4	c.89200+4 T>C	Cryptic splicing	296	100	0.00023870	A‐band	△score < 0.20	VUS	HCM	—	[25]
25	NM_133378.4	c.98670+4A>G	Cryptic splicing	307	100	NA	Ig‐like 149 (A‐band)	△score < 0.20	LB	HCM	—	[26]
26	NM_133437	c.9977‐4G>A	Cryptic splicing	42	100	0.00008225	Ig‐like 209 (I‐band)	△score < 0.20	LB	HCM	*DSP*:c.1_2insA	[24]
27	NM_133437	c.43097‐5C>G	Cryptic splicing	154	100	0.00373400	FN‐III 57 (I‐band)	△score < 0.20	LB	HCM	—	[24]
28	NM_133437	c.70574‐3T>C	Cryptic splicing	177	100	0.00018980	FN‐III 124 (I‐band)	△score < 0.20	VUS	HCM	*SCN5A*: p.D1819N	[24]

Abbreviations: B, benign; FAF, filtering allele frequency; FN‐III, fibronectin type‐III; HCM, hypertrophic cardiomyopathy; Ig‐like, immunoglobulin (Ig)‐like domain; LB, likely benign; LQTS, long QT syndrome; LVOTO, left ventricular outflow tract obstruction; NA, not available; NSVT, nonsustained ventricular tachycardia; PEVK, the proline‐, glutamate‐, valine‐, and lysine‐rich; PSI, percentage spliced in; SIDS, sudden infant death syndrome; SUD, sudden unexplained death; VUS, variant of uncertain significance.

## 5. Discussion

To our knowledge, this study described the first Chinese pedigree with HCM associated with the homozygous *TTN* variant. Functional analysis via the minigene splicing assay revealed that cryptic splice‐site activation induced the in‐frame indels (p.Arg32498_Glu32504delinsGln). *TTN* variants have been reported in various cardiomyopathies including HCM, LVNC, and ARVC [13–16]. Although *TTN* has definitive gene‐disease validity for DCM, its role in HCM remains controversial due to limited evidence. Since the initial report linking *TTN* and HCM, TTNtv have been detected in 1.3%–4% of HCM cases [13, 15, 24, 27]. Some variants in *TTN* were reported in sporadic individuals with HCM. For instance, a heterozygous missense variant (rs201381085) was identified in a patient with nonobstructive HCM [28]. In a cohort of 242 HCM patients, a TTNtv (c.70437delG) located in the A‐band was detected in one patient, classified as LP, and categorized as a non–HCM‐associated variant [29]. Additionally, a pathogenic splice‐site variant (c.22816+1G>T) near the I‐band terminus was documented in a Romanian HCM patient [21]. However, case‐control studies have failed to provide compelling evidence for a strong association between *TTN* and HCM. This is because *TTN* variants are detected in both patient and control groups, with no significant enrichment of *TTN* variants in the case cohorts [13, 15]. These conflicting results challenge the association between the *TTN* gene and HCM and suggest that this relationship needs further investigations.

TTNtv represent a prominent genetic alteration in cardiomyopathies [13, 15]. Although canonical splice‐site variants are often grouped with truncating variants when they are predicted to cause frameshift or nonsense‐mediated decay, the variant reported here activates a cryptic acceptor site and produces an in‐frame indel. Several variants were reclassified as LB or B due to high population frequencies, thereby excluding their potential pathogenicity. The remaining variants demonstrated insufficient evidence for classification as LP or higher. Most identified variants localized to the I‐band region, affecting exons within low PSI transcripts in myocardial tissue [12]. Furthermore, several splicing variants situated within the A‐band region were predicted to induce in‐frame exon skipping, thereby preserving the reading frame, and not resulting in a frameshift. Compared with many reported *TTN* splicing variants in HCM, which either affect low‐PSI exons, lack RNA validation, or co‐occur with pathogenic variants in established sarcomeric genes, the present variant affects a highly included A band exons and produces a minigene‐confirmed in‐frame indel. The minigene result predicts preservation of the reading frame and does not support a classical truncating mechanism in this assay. Although the aberrant transcript is predicted to preserve the reading frame, we did not assess endogenous *TTN* transcript abundance, titin protein expression, protein stability, sarcomere incorporation, or binding to interacting partners. Therefore, we cannot exclude altered protein stability or functional impairment of the affected titin domain. Specific in‐frame deletions affecting functional amino acid motifs in A‐band domains may exhibit HCM association potential, though this hypothesis requires validation through expanded cohort analyses and mechanistic functional studies.

The inheritance pattern of HCM is primarily AD. However, variants in *TTN* and other genes typically associated with dominant inheritance can also manifest through recessive mechanisms. In the present study, familial analysis revealed autosomal recessive (AR) inheritance. Although rare, homozygous variants in sarcomeric genes are associated with severe cardiac phenotypes. For instance, a homozygous *MYBPC3* in‐frame deletion (chr11:g.47357432_47357458del) has been reported to result in combined HCM/DCM phenotypes [30], and a pathogenic *MYH7* in‐frame deletion (c.2791_2793delGAG) has been linked to infantile‐onset HCM [31]. A novel homozygous *TPM1* variant (p.Gly3Arg) has been identified as a cause of AR pediatric HCM [32].

Rare *TTN* variants have previously been reported in sporadic HCM cases [21, 28, 29]. These reports provide context for considering *TTN* variants in HCM. In the present family, the proband was homozygous for a splice‐altering *TTN* variant predicted to preserve the reading frame. The heterozygous offspring were clinically asymptomatic at their current ages. This observation is compatible with a possible dosage‐dependent or recessive model. We hypothesize that a subset of rare HCM cases may result from homozygous variants, which are transmitted through AR inheritance within families. Therefore, our findings raise the possibility that homozygosity for *TTN* variants predicted to retain partial protein function may contribute to HCM through AR inheritance. Such variants could include missense variants, small in‐frame deletions, or splice‐altering variants that preserve the reading frame. The residual functional capacity preserved in such variants might account for their recessive inheritance pattern while still maintaining sufficient pathogenicity to manifest clinical phenotypes under homozygous conditions.

Furthermore, previous studies utilizing mouse models carrying *TTN* variants have demonstrated HCM phenotypes. Specifically, adult homozygous PEVK knockout mice exhibited diastolic dysfunction and cardiac hypertrophy accompanied by upregulation of FHL proteins [33]. Similarly, titin M‐line–deficient mice developed cardiac hypertrophy and heart failure [34]. Although the relationship between *TTN* and HCM remains classified as limited in the recent ClinGen, the evidence score was close to the threshold for moderate, with the final classification remaining limited mainly because of insufficient case‐level evidence [8]. Several functional studies nevertheless support the biological plausibility of *TTN* involvement in HCM. For example, variant p.Arg740Leu in *TTN* was demonstrated to increase the binding affinity of titin to alpha‐actinin in a yeast two‐hybrid assay [27]. Investigating novel pathogenic pathways, a study identified a missense variant in an Ig domain near the M‐line–A‐band transition zone of *TTN* in a medaka mutagenesis fish model exhibiting diastolic dysfunction [35]. Additionally, the authors of this study identified two missense variants in titin′s Ig domains in patients with HCM and provided functional evidence that these variants enhance binding to muscle‐specific ring finger protein 1 (MURF1), leading to increased titin degradation through ubiquitination [35].

Current variant detection methodologies likely underestimate exonic and noncanonical splicing alterations, as comprehensive RNA analyses remain technically challenging in clinical practice. Minigene assays offer a practical method for *in vitro* splicing analysis. Functional verification confirmed that this variant conforms to the PM4 standard, suggesting that it be reclassified as a tepid VUS (scoring 3 points). Consistent with current ESC consensus on cardiovascular genomics, warm/hot VUS carriers require management analogous to LP variants [36]. However, because the present variant remains a tepid VUS after functional verification, clinical management in this family should be driven primarily by the proband′s phenotype, the inheritance model, and continued familial reassessment.

*TTN* variants have been reported in patients with early‐onset AF. However, the strongest evidence in AF relates mainly to heterozygous truncating or loss‐of‐function variants located in highly expressed cardiac exons. In the UK Biobank study, high‐PSI *TTN* loss‐of‐function variants were associated with AF, but AF was present in only 14% of carriers, and polygenic risk substantially modified penetrance [37]. Similarly, studies of early‐onset AF have shown enrichment of pathogenic/LP variants in cardiomyopathy genes, with *TTN* being common among positive findings, but most such variants were interpreted in the broader context of inherited cardiomyopathy rather than isolated AF [38]. In cohorts of TTNtv carriers, AF is frequently observed together with, or in the setting of progression toward, left ventricular dysfunction; although AF may occasionally precede overt LV systolic dysfunction, this does not exclude latent or evolving cardiomyopathy [39]. A recent study of AF patients with pathogenic *TTN* variants also focused on high‐PSI truncating variants and found that nearly half‐developed LV systolic dysfunction and/or malignant ventricular arrhythmias during follow‐up [40]. Although the literature supports TTNtv as important contributors to AF susceptibility, particularly in early‐onset AF and AF associated with cardiomyopathy, these data do not directly establish that a nontruncating homozygous splice‐site variant such as the one reported here is primarily an AF‐only Mendelian cause. Furthermore, major gene‐disease curation resources, including ClinGen, GenCC, Gene2Phenotype, and Morbid, have not systematically curated a Mendelian association between *TTN* and isolated AF. This absence of formal curation further supports a cautious interpretation: Rare *TTN* variants are credible contributors to AF risk, especially when embedded within a cardiomyopathy phenotype, but they do not yet fulfill the evidentiary criteria for a standalone Mendelian AF gene. In this context, the present case may contribute additional clinical and functional evidence to this evolving gene–disease relationship.

The homozygous splice‐site variant identified here affects a highly cardiac‐included *TTN* exon and was shown by minigene assay to generate an in‐frame deletion/insertion, suggesting a potential mechanism by which disruption of specific cardiac *TTN* exons, particularly in a biallelic state, may contribute to HCM or an HCM‐arrhythmia overlap phenotype. We interpreted the variant as a candidate contributor to the patient′s cardiomyopathy‐centered phenotype, with AF/flutter potentially representing part of a broader cardiomyopathy‐arrhythmia presentation rather than definitive evidence for an isolated AF mechanism.

### 5.1. Limitations

*TTN* is characterized by highly complex and tissue‐specific alternative splicing, and minigene assays cannot fully recapitulate the endogenous regulatory context of *TTN* pre‐mRNA processing in human cardiomyocytes. Therefore, patient‐derived RNA analysis would, in principle, provide more direct evidence for the endogenous splicing consequence of this variant. However, the feasibility and interpretability of blood‐based RNA analysis for *TTN* are limited by both tissue expression and exon‐level splicing context. *TTN* is expressed predominantly in striated muscle, and its transcript abundance in peripheral blood is very low. In the available blood transcriptomic data, the expression level of the relevant *TTN* region was low (TPM = 0.2919), suggesting that peripheral blood RNA would have limited sensitivity for detecting aberrant splicing at this locus. In addition, because *TTN* has an exceptionally large transcript size and complex isoform structure, short‐read RNA sequencing from a low‐expression tissue may provide uneven coverage and may not reliably resolve the relevant full‐length splicing event. Thus, even if blood RNA were available, a negative result would not necessarily exclude an endogenous splicing defect in cardiac tissue.

Unfortunately, we were unable to obtain an additional blood sample from the patient for RNA analysis. DNA samples from the proband′s parents and siblings were not available. Parental carrier status and broader familial segregation could not be assessed. Future research aimed at fully characterizing the role of *TTN* variants in HCM and developing novel diagnostics and therapeutics will probably require integrating multiomics functional analyses with studies of larger cohorts.

## 6. Conclusion

To our knowledge, this study is the first to describe a Chinese pedigree with HCM linked to a homozygous *TTN* variant. The minigene assay demonstrated that this deletion caused aberrant pre‐mRNA splicing, resulting in the complex in‐frame indels (c.97493_97510del; p.Arg32498_Glu32504delinsGln) within the FN III domain. This alteration may be related to the less severe HCM phenotype. While describing a single variant cannot fully elucidate the correlation between genotype and phenotype, the results of this study provide new insights into the molecular genetic mechanisms underlying HCM.

## Author Contributions

Xiaoyun Sun and Dandan Wang carried out all the data analyses, participated in the design of the work, and wrote the original draft. Xiaoyun Sun and Rong Wan designed the experiments. Shujuan Wang, Zhenhong Jiang, and Jin Ouyang participated in molecular genetic test. Wenqiang Zhang and Jin Ouyang participated in data analyses. Yang Shen and Kui Hong reviewed and revised manuscripts and provided financial support. Xiaoyun Sun and Dandan Wang contributed equally.

## Funding

This study was supported by the National Natural Science Foundation of China (No. 82360048), Jiangxi Province Natural Science Foundation (20223BBG71011), and the Molecular Medicine Jiangxi Provincial Key Laboratory Support Program (2024SSY06231).

## Disclosure

All authors read and approved the final manuscript.

## Ethics Statement

This study was approved by the Medical Ethics Committee of The Second Affiliated Hospital of Nanchang University. The informed consent from the patient′s parents prior to conducting the genetic testing had been obtained.

## Conflicts of Interest

The authors declare no conflicts of interest.

## Supporting information


**Supporting Information** Additional supporting information can be found online in the Supporting Information section. Method S1 (minigene assay): To create hybrid minigene constructs, we used the pcMINI vector that we developed previously. We cloned DNA fragments containing a couple of exons and introns around the target variant in the TTN gene using classical restriction enzyme digestion and ligation methods. We described the detailed steps for constructing the mini gene carrying the c.97493‐2_97504del variant. Figure S1: The results of in silico splice‐site predictions. (A) SpliceAI predicts that this variant the disruption of the original acceptor site, and the creation of a new acceptor site may affect splicing (△score 0.99); (B) RDDC^SC^ predicts that the disruption of the original acceptor site, and the creation of a new acceptor site may affect splicing; (C) VarSEAK predicts that the original acceptor site is disrupted, creating a new acceptor site, and that the confidence score is elevated by 74.93%, affecting the results of splicing. Table S1: Primer sequences in the minigene constructs carrying the c.97493‐2_97504del variant. Table S2: Cardiomyopathy‐ and inherited arrhythmia‐associated genes prioritized from WES.

## Data Availability

Raw sequencing data are not publicly available due to privacy or ethical restrictions but may be provided by the corresponding author upon reasonable request, where appropriate and subject to approval.
