# Supplementary material for: Functional Validation of a Novel Homozygous TTN Splice‐Site Variant Reveals Aberrant Splicing in Hypertrophic Cardiomyopathy
Source: Hum Mutat. 2026 Sep 3;2026:4229370. doi: 10.1155/humu/4229370 (PMC13542574; doi:10.1155/humu/4229370)
Supplement: Supplementary file 1 [file HUMU-2026-4229370-s001.docx]

Supplementary Materials

# Supplementary method

**Minigene assay**:

To create hybrid minigene constructs, we used the pcMINI vector that we developed previously. We cloned DNA fragments containing a couple of exons and introns around the target variant in the *TTN* gene using classical restriction enzyme digestion and ligation methods. Detailed steps for minigene constructs carrying the c.97493-2_97504del variant were as follows:

(1) Two pairs of nested primers (261546-*TTN*-F with 265093-*TTN*-R and 261838-*TTN*-F with 264821-*TTN*-R) were designed. Nested PCR was carried out with normal human genomic DNA as the template.

(2) Wild-type fragment amplification: the second round of nested PCR was used as the template and pcMINI-N-*TTN*-EcoRI-F and pcMINI-N-*TTN*-XhoI-R were used as primers to amplify the 1945bp wild‐type fragment of pcMINI.

(3) Mutant fragment amplification: pcMINI-N-*TTN*-EcoRI-F with *TTN*-mut-R and pcMINI-N-*TTN*-XhoI-R with *TTN*-mut-F were used as primers to amplify the upstream and downstream segments, respectively. Equimolar mixtures of these segments were used as template with flanking primers (pcMINI-N-*TTN*-EcoRI-F and pcMINI-N-*TTN*-XhoI-R) to generate the 1945-bp mutant fragment.

The hybrid minigenes were confirmed by sequencing and transfected into HEK293T and HeLa cells. After 48 hours, total RNA was extracted using the RNA extraction kit (Trizol RNAiso PLUS, 9109, TaKaRa), reverse-transcribed with HifairTM 1st Strand cDNA Synthesis SuperMix (11123ES70, YEASEN), and PCR-amplified. Amplification products were resolved on 1.5% agarose gels and validated by Sanger sequencing.

**Supplementary Figure 1** The results of in silico splice site predictions

(A) SpliceAI predicts that this variant the disruption of the original Acceptor site and the creation of a new Acceptor site may affect splicing (△score 0.99); (B) RDDC^SC^ predicts that the disruption of the original Acceptor site and the creation of a new Acceptor site may affect splicing; (C) Varseak predicts that the original Acceptor site is disrupted, creating a new Acceptor site, and that the confidence score is elevated by 74.93%, affecting the results of splicing.

**Supplementary Table 1**

Primer sequences in the minigene constructs carrying the c.97493-2_97504del variant.

| **Primer name** | Primer sequence (5´‐ 3´) |
| --- | --- |
| 261546-TTN-F | CACAGCCGTGACTGTACAAG |
| 261838-TTN-F | CAGAGCGTGTCACAGTTGAA |
| 264821-TTN-R | CGCTTACTAATATCCTGGCC |
| 265093-TTN-R | GCACTTCTCGTCTCTCGAGG |
| TTN-mut-F | cctctgtcttgctgcCCCCAGAAACATTAC |
| TTN-mut-R | GTAATGTTTCTGGGGgcagcaagacagagg |
| pcMINI-N-TTN-EcoRI-F | GGTGGAATTCatggACAAGCCTGGTCCACCAACA |
| pcMINI-N-TTN-XhoI-R | tttcCTCGAGaaaattagccatgcgtggtg |

# Supplementary Table 2. Cardiomyopathy- and inherited arrhythmia-associated genes prioritized from WES.

Whole-exome sequencing was performed, and variant prioritization focused on a virtual gene set of cardiomyopathy- and inherited arrhythmia-associated genes with ClinGen-curated or ClinGen-based gene-disease validity classifications of Limited or higher. Gene-disease pairs are listed separately when the same gene had different evidence classifications across phenotypes.

| **Disease group** | **Gene** | **Disease/phenotype evaluated** | **Gene-disease validity classification** |
| --- | --- | --- | --- |
| Hypertrophic cardiomyopathy / LVH phenocopies | *MYBPC3* | HCM | Definitive |
| Hypertrophic cardiomyopathy / LVH phenocopies | *MYH7* | HCM | Definitive |
| Hypertrophic cardiomyopathy / LVH phenocopies | *TNNT2* | HCM | Definitive |
| Hypertrophic cardiomyopathy / LVH phenocopies | *TNNI3* | HCM | Definitive |
| Hypertrophic cardiomyopathy / LVH phenocopies | *TNNC1* | HCM | Definitive |
| Hypertrophic cardiomyopathy / LVH phenocopies | *TPM1* | HCM | Definitive |
| Hypertrophic cardiomyopathy / LVH phenocopies | *ACTC1* | HCM | Definitive |
| Hypertrophic cardiomyopathy / LVH phenocopies | *MYL2* | HCM | Definitive |
| Hypertrophic cardiomyopathy / LVH phenocopies | *MYL3* | HCM | Definitive |
| Hypertrophic cardiomyopathy / LVH phenocopies | *ACTN2* | Intrinsic cardiomyopathy | Definitive |
| Hypertrophic cardiomyopathy / LVH phenocopies | *ALPK3* | Infant-onset HCM/DCM | Definitive |
| Hypertrophic cardiomyopathy / LVH phenocopies | *CSRP3* | HCM | Definitive |
| Hypertrophic cardiomyopathy / LVH phenocopies | *FHOD3* | HCM | Definitive |
| Hypertrophic cardiomyopathy / LVH phenocopies | *PLN* | Intrinsic cardiomyopathy | Definitive |
| Hypertrophic cardiomyopathy / LVH phenocopies | *CACNA1C* | Timothy syndrome | Definitive |
| Hypertrophic cardiomyopathy / LVH phenocopies | *DES* | Desminopathy | Definitive |
| Hypertrophic cardiomyopathy / LVH phenocopies | *FHL1* | Emery-Dreifuss muscular dystrophy | Definitive |
| Hypertrophic cardiomyopathy / LVH phenocopies | *FLNC* | Myofibrillar myopathy | Definitive |
| Hypertrophic cardiomyopathy / LVH phenocopies | *GLA* | Fabry disease | Definitive |
| Hypertrophic cardiomyopathy / LVH phenocopies | *LAMP2* | Danon disease | Definitive |
| Hypertrophic cardiomyopathy / LVH phenocopies | *PRKAG2* | Cardiomyopathy | Definitive |
| Hypertrophic cardiomyopathy / LVH phenocopies | *TTR* | Transthyretin amyloidosis | Definitive |
| Hypertrophic cardiomyopathy / LVH phenocopies | *PTPN11* | Noonan syndrome | Definitive |
| Hypertrophic cardiomyopathy / LVH phenocopies | *RAF1* | Noonan syndrome | Definitive |
| Hypertrophic cardiomyopathy / LVH phenocopies | *RIT1* | Noonan syndrome | Definitive |
| Hypertrophic cardiomyopathy / LVH phenocopies | *ALPK3* | HCM | Strong |
| Hypertrophic cardiomyopathy / LVH phenocopies | *JPH2* | HCM | Moderate |
| Hypertrophic cardiomyopathy / LVH phenocopies | *KLHL24* | HCM | Moderate |
| Hypertrophic cardiomyopathy / LVH phenocopies | *TRIM63* | HCM | Moderate |
| Hypertrophic cardiomyopathy / LVH phenocopies | *MT-TI* | HCM | Moderate |
| Hypertrophic cardiomyopathy | *TTN* | HCM | Limited |
| Hypertrophic cardiomyopathy | *RYR2* | HCM | Limited |
| Hypertrophic cardiomyopathy | *OBSCN* | HCM | Limited |
| Hypertrophic cardiomyopathy | *KLF10* | HCM | Limited |
| Hypertrophic cardiomyopathy | *RPS6KB1* | HCM | Limited |
| Hypertrophic cardiomyopathy | *PDLIM3* | HCM | Limited |
| Hypertrophic cardiomyopathy | *NEXN* | HCM | Limited |
| Hypertrophic cardiomyopathy | *RBM20* | HCM | Limited |
| Hypertrophic cardiomyopathy | *TMPO* | HCM | Limited |
| Dilated cardiomyopathy | *BAG3* | Dilated cardiomyopathy | Definitive |
| Dilated cardiomyopathy | *DES* | Dilated cardiomyopathy | Definitive |
| Dilated cardiomyopathy | *FLNC* | Dilated cardiomyopathy | Definitive |
| Dilated cardiomyopathy | *LMNA* | Dilated cardiomyopathy | Definitive |
| Dilated cardiomyopathy | *MYH7* | Dilated cardiomyopathy | Definitive |
| Dilated cardiomyopathy | *PLN* | Dilated cardiomyopathy | Definitive |
| Dilated cardiomyopathy | *RBM20* | Dilated cardiomyopathy | Definitive |
| Dilated cardiomyopathy | *SCN5A* | Dilated cardiomyopathy | Definitive |
| Dilated cardiomyopathy | *TNNC1* | Dilated cardiomyopathy | Definitive |
| Dilated cardiomyopathy | *TNNT2* | Dilated cardiomyopathy | Definitive |
| Dilated cardiomyopathy | *TTN* | Dilated cardiomyopathy | Definitive |
| Dilated cardiomyopathy | *DSP* | Dilated cardiomyopathy | Strong |
| Dilated cardiomyopathy | *ACTC1* | Dilated cardiomyopathy | Moderate |
| Dilated cardiomyopathy | *ACTN2* | Dilated cardiomyopathy | Moderate |
| Dilated cardiomyopathy | *JPH2* | Dilated cardiomyopathy | Moderate |
| Dilated cardiomyopathy | *NEXN* | Dilated cardiomyopathy | Moderate |
| Dilated cardiomyopathy | *TNNI3* | Dilated cardiomyopathy | Moderate |
| Dilated cardiomyopathy | *TPM1* | Dilated cardiomyopathy | Moderate |
| Dilated cardiomyopathy | *VCL* | Dilated cardiomyopathy | Moderate |
| Dilated cardiomyopathy | *ABCC9* | Dilated cardiomyopathy | Limited |
| Dilated cardiomyopathy | *ANKRD1* | Dilated cardiomyopathy | Limited |
| Dilated cardiomyopathy | *CSRP3* | Dilated cardiomyopathy | Limited |
| Dilated cardiomyopathy | *CTF1* | Dilated cardiomyopathy | Limited |
| Dilated cardiomyopathy | *DSG2* | Dilated cardiomyopathy | Limited |
| Dilated cardiomyopathy | *DTNA* | Dilated cardiomyopathy | Limited |
| Dilated cardiomyopathy | *EYA4* | Dilated cardiomyopathy | Limited |
| Dilated cardiomyopathy | *GATAD1* | Dilated cardiomyopathy | Limited |
| Dilated cardiomyopathy | *ILK* | Dilated cardiomyopathy | Limited |
| Dilated cardiomyopathy | *LAMA4* | Dilated cardiomyopathy | Limited |
| Dilated cardiomyopathy | *LDB3* | Dilated cardiomyopathy | Limited |
| Dilated cardiomyopathy | *MYBPC3* | Dilated cardiomyopathy | Limited |
| Dilated cardiomyopathy | *MYH6* | Dilated cardiomyopathy | Limited |
| Dilated cardiomyopathy | *MYL2* | Dilated cardiomyopathy | Limited |
| Dilated cardiomyopathy | *MYPN* | Dilated cardiomyopathy | Limited |
| Dilated cardiomyopathy | *NEBL* | Dilated cardiomyopathy | Limited |
| Dilated cardiomyopathy | *NKX2-5* | Dilated cardiomyopathy | Limited |
| Dilated cardiomyopathy | *OBSCN* | Dilated cardiomyopathy | Limited |
| Dilated cardiomyopathy | *PLEKHM2* | Dilated cardiomyopathy | Limited |
| Dilated cardiomyopathy | *PRDM16* | Dilated cardiomyopathy | Limited |
| Dilated cardiomyopathy | *PSEN2* | Dilated cardiomyopathy | Limited |
| Dilated cardiomyopathy | *SGCD* | Dilated cardiomyopathy | Limited |
| Dilated cardiomyopathy | *TBX20* | Dilated cardiomyopathy | Limited |
| Dilated cardiomyopathy | *TCAP* | Dilated cardiomyopathy | Limited |
| Dilated cardiomyopathy | *TNNI3K* | Dilated cardiomyopathy | Limited |
| Arrhythmogenic right ventricular cardiomyopathy | *PKP2* | Classic ARVC | Definitive |
| Arrhythmogenic right ventricular cardiomyopathy | *DSP* | ARVC, frequent left ventricular involvement | Definitive |
| Arrhythmogenic right ventricular cardiomyopathy | *DSG2* | ARVC, frequent left ventricular involvement | Definitive |
| Arrhythmogenic right ventricular cardiomyopathy | *DSC2* | ARVC | Definitive |
| Arrhythmogenic right ventricular cardiomyopathy | *JUP* | Naxos disease | Definitive |
| Arrhythmogenic right ventricular cardiomyopathy | *TMEM43* | ARVC subtype with high sudden-death risk in males | Definitive |
| Arrhythmogenic right ventricular cardiomyopathy | *PLN* | ARVC, frequent left ventricular involvement | Moderate |
| Arrhythmogenic right ventricular cardiomyopathy | *DES* | ARVC, conduction system abnormalities common | Moderate |
| Arrhythmogenic right ventricular cardiomyopathy | *SCN5A* | ARVC | Limited |
| Arrhythmogenic right ventricular cardiomyopathy | *LMNA* | ARVC | Limited |
| Arrhythmogenic right ventricular cardiomyopathy | *CDH2* | ARVC | Limited |
| Arrhythmogenic right ventricular cardiomyopathy | *CTNNA3* | ARVC | Limited |
| Arrhythmogenic right ventricular cardiomyopathy | *TGFB3* | ARVC | Limited |
| Arrhythmogenic right ventricular cardiomyopathy | *TTN* | ARVC | Limited |
| Arrhythmogenic right ventricular cardiomyopathy | *TJP1* | ARVC | Limited |
| Arrhythmogenic right ventricular cardiomyopathy | *MYH7* | ARVC | Limited |
| Arrhythmogenic right ventricular cardiomyopathy | *MYBPC3* | ARVC | Limited |
| Arrhythmogenic right ventricular cardiomyopathy | *MYL3* | ARVC | Limited |
| Long QT syndrome | *KCNH2* | LQTS | Definitive |
| Long QT syndrome | *KCNQ1* | LQTS | Definitive |
| Long QT syndrome | *SCN5A* | LQTS | Definitive |
| Long QT syndrome | *CALM1* | LQTS with atypical features | Definitive |
| Long QT syndrome | *CALM2* | LQTS with atypical features | Definitive |
| Long QT syndrome | *CALM3* | LQTS with atypical features | Definitive |
| Long QT syndrome / syndromic arrhythmia | *CACNA1C* | Timothy syndrome | Definitive |
| Long QT syndrome / syndromic arrhythmia | *KCNJ2* | Andersen-Tawil syndrome | Definitive |
| Long QT syndrome | *TRDN* | LQTS with atypical features | Strong |
| Long QT syndrome | *CACNA1C* | LQTS | Moderate |
| Long QT syndrome | *CAV3* | LQTS | Limited |
| Long QT syndrome | *KCNE1* | LQTS | Limited |
| Long QT syndrome | *KCNJ2* | LQTS | Limited |
| Acquired long QT syndrome | *KCNE1* | Acquired LQTS risk-allele context | Strong |
| Acquired long QT syndrome | *KCNE2* | Acquired LQTS risk-allele context | Strong |
| Catecholaminergic polymorphic ventricular tachycardia | *RYR2* | CPVT | Definitive |
| Catecholaminergic polymorphic ventricular tachycardia | *CASQ2* | CPVT | Definitive |
| Catecholaminergic polymorphic ventricular tachycardia | *CASQ2* | CPVT | Moderate |
| Catecholaminergic polymorphic ventricular tachycardia | *TRDN* | CPVT | Definitive |
| Catecholaminergic polymorphic ventricular tachycardia | *TECRL* | CPVT | Definitive |
| Catecholaminergic polymorphic ventricular tachycardia | *CALM1* | CPVT | Moderate |
| Catecholaminergic polymorphic ventricular tachycardia | *CALM2* | CPVT | Moderate |
| Catecholaminergic polymorphic ventricular tachycardia | *CALM3* | CPVT | Moderate |
| Short QT syndrome | *KCNH2* | SQTS | Definitive |
| Short QT syndrome | *KCNQ1* | SQTS | Strong |
| Short QT syndrome | *KCNJ2* | SQTS | Moderate |
| Short QT syndrome | *SLC4A3* | SQTS | Moderate/Strong |
